# Ionic Liquid assisted Synthesis of Zeolite-TON

**DOI:** 10.1002/zaac.201400103

**Published:** 2014-03-20

**Authors:** Yuyang Tian, Matthew J McPherson, Paul S Wheatley, Russell E Morris

**Affiliations:** [a] EaSt Chem. School of Chemistry, University of St Andrews Purdie Building, St Andrews KY16 9ST, UK

**Keywords:** Zeolites, Synthetic methods, Solid-phase synthesis, Ionic liquids, Green chemistry

## Abstract

An ionic liquid assisted strategy for the synthesis of zeolitic material is reported. This strategy is a solid state synthetic method and the ionic liquid is employed as structure directing agent. A TON-type zeolite, which contains one-dimensional 10-member-ring, is successfully synthesized with the assistance of the ionic liquid, 1-ethyl-3-methylimidazolium bromide. This finding improves our understanding about the challenge of ionothermally synthesizing siliceous and aluminosilicate zeolites.

## Introduction

Since the pioneering discoveries of synthesizing zeolites under hydrothermal conditions,[1,2] many researches on the zeolite preparation of both naturally existed analogues and new zeolitic structures have been reported.[3–7] The enthusiasm for synthesizing zeolites is driven by their diverse applications such as ion-exchange,[8–11] adsorption,[12–16] and catalysis.[17–19] From the beginning, the hydrothermal route is the preferred method of synthesizing zeolites, in which the source of tetrahedral atoms and templates are heated in basic aqueous solution at temperatures above 100°C under autogenous pressure. In later years other synthetic routes were developed: The solvothermal synthesis route,[20,21] which utilizes organic solvents as reaction media, is proved to be successful in zeolite synthesis, especially in the preparation of large single crystals[22] and unprecedented structures that are never obtained from hydrothermal routes;[23] Dry Gel Conversion (DGC) is a method for the synthesis of aluminosilicate and pure silica zeolites.[24,25] In the DGC route amorphous dry gel that contains zeolite precursor is crystallized by the assistance of steam or volatile organic amines.[26] Another novel synthetic route for the zeolitic analogues is ionothermal synthesis, which employ ionic liquids as solvent and template at the same time.[27] Ionic liquids are a class of organic molten salts with melting points under 100 °C.[28,29] The compounds consist of only ions and possess negligible vapor pressure, which reduces the safety concern of explosion for reaction in sealed autoclaves.[30] Other potential advantages of ionothermal route also include the improved templating effect, which is caused by the absence of competition between solvent and template for their interaction with tetrahedral atoms,[31] and also the designable organic ions may direct to a series of new structures.[32]

Mechanochemical synthesis is considered to be an effective and sustainable synthetic method and has attracted increasing attention.[33] Numerous materials such as inorganic compounds, organic compounds, and hybrid coordinated polymers were successfully synthesized by mechanically mixing and grinding the starting solids.[34] Zeolite materials were also obtained by mechanochemical reaction following hydrothermal treatment.[35–37]

Recently, a novel solvent free synthetic approach has been reported by *Ren* et al.[38] In this approach, tetrahedral atom precursors and structure directing agents (SDAs) are mechanically mixed and heated in an autoclave without addition of any other solvent. Several zeolite structure types including MFI, BEA, MOR, and FAU are successfully prepared with minimal waste solvent produced. This strategy is considered to be a “green chemistry” procedure because of high yields and the diminution of produced waste. Inorganic or organic cations are used as SDAs in the procedure.

Similar to organic quaternary ammonium compounds,[39] commonly used ionic liquids contain organic cations which can play the role of SDAs.[31] Replacing the common SDAs with ionic liquids for zeolite synthesis may be advantageous by reducing the autogenous pressure and the possibility of obtaining some new pore architectures due to the unique properties of ionic liquids.

Up to now many porous materials including aluminophosphates (AlPOs)[32,40] and metal organic frameworks (MOFs)[41,42] are synthesized from the ionothermal synthetic routes. However there is still a big challenge in ionothermally synthesizing siliceous and aluminosilicate zeolites, which are important catalysts in petrochemistry industry.[43,44] This is possibly owing to the poor solubility of silica species in commonly used ionic liquids and the lack of mineralizers in the ionothermal system. Meanwhile some ionic liquids can reduce the hydrolysis activity of water.[45,46] Only a few work reported the synthesis of siliceous zeolites with the aid of ionic liquids.[47–50] *Wheatley* et al.[49] reported the synthesis of siliceous zeolites in the partially hydroxide anion-exchanged ionic liquid. This task-specific ionic liquid contains hydroxide anions which are constructive for the dissolution of silicate precursor. *Yan* and co-workers[50] prepared zeolite MFI using ionic liquid under microwave heating. Although the mixture of water/ionic liquid is used, the reaction still occurred under ambient pressure due to the assistance of ionic liquid.

**Figure 1:**
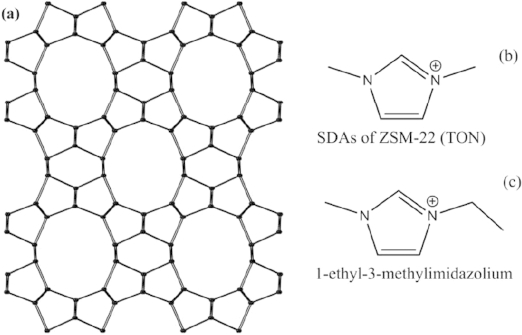
(a) TON type zeolite structure viewed along the 10-member ring channels. (b) Reported SDA structure for the synthesis of ZSM-22.[52] (c) Structure of the ionic liquid for the ionic liquid assisted synthesis of TON zeolite in this work.

In this work one of the most commonly used ionic liquids, 1-ethyl-3-methylimidazolium bromide (EmimBr), is employed in the solvent free synthetic procedure. The aim is to produce siliceous or aluminosilicate zeolites with the aid of ionic liquids. Powder X-ray diffraction (PXRD) identified the product as TON structure, which is a one-dimensional zeolite with 10-member-ring channels (Figure1a).[51] Aluminum species was found to be necessary for the zeolite formation. Scanning electron microscopy (SEM), solid state NMR, thermogravimetric analysis (TGA), and nitrogen adsorption were applied to characterize the products. Such a synthetic route which we name as ionic liquid assisted (ILA) synthesis improves our understanding about ionothermally synthesizing siliceous and aluminosilicate zeolites.

## Results and Discussions

**Figure 2:**
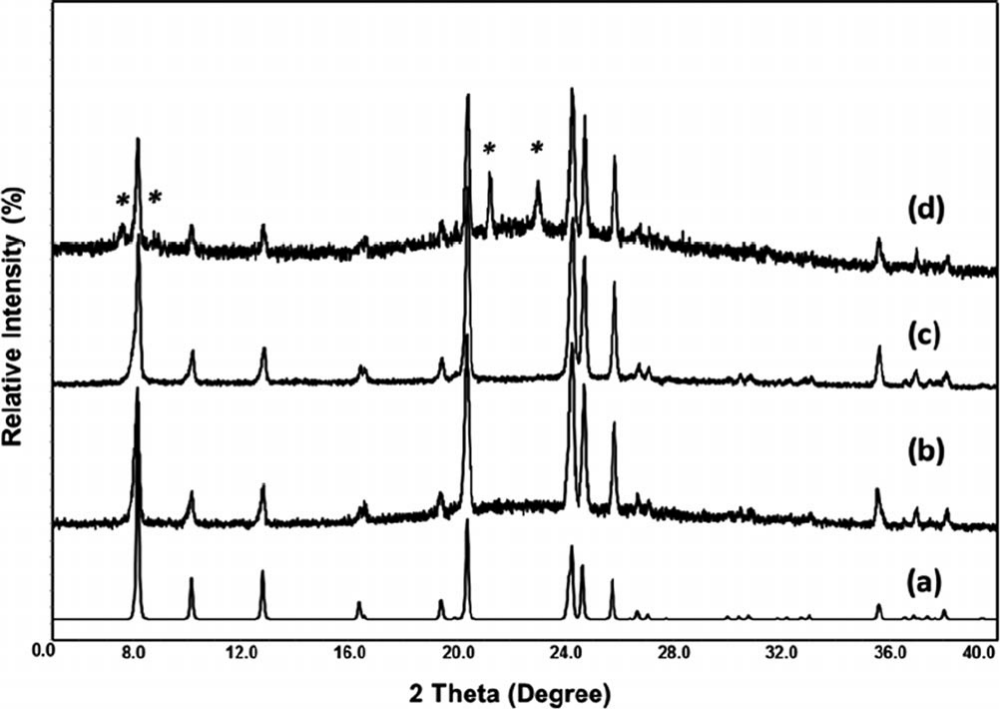
PXRD patterns of (a) simulated TON type structure; (b) as-made pure TON sample (No. 7); (c) as-made pure TON sample synthesized with addition of water (No. 8) and (d) as-made TON sample which contains impurity of *MRE (No. 2, the asterisks indicate the reflections from the impurity).

The synthetic compositions and the products are listed in Table 1. A systematic study on the effect of the contents of the aluminium precursor, SDA and water were carried out. In general, those synthetic compounds have the chemical compositions of 0.5Na_2_O:1SiO_2_:0.7NH_4_Cl:*x*Al_2_O_3_:*y*EmimBr:*z*H_2_O. For the samples of No.1, 2, 4–7, and 9–12, the water only comes from the starting material of Na_2_SiO_3_**·**9H_2_O. The ratio of H_2_O/SiO_2_ is 4.5. For the samples of No. 3 and 8, which are aimed to examine the effect of operation environment, small amount of water is added into the compounds and the H_2_O/SiO_2_ ratio increases to 5. The structures of the products are identified by PXRD, which are shown in Figure2.

**Table 1:** Experimental details of ILA synthetic routea)

No.	*x*Al_2_O_3_	*y*EmimBr	*z*H_2_O	Products
1	0	0	4.5	α-quartz
2	0	0.1	4.5	TON+^*^MRE
3	0	0.2	5	TON+^*^MRE
4	0.006	0	4.5	α-quartz
5	0.006	0.1	4.5	TON
6	0.013	0	4.5	α-quartz
7	0.013	0.1	4.5	TON
8	0.013	0.2	5	TON
9	0.025	0	4.5	α-quartz
10	0.025	0.1	4.5	TON
11	0.037	0	4.5	amorphous
12	0.037	0.1	4.5	amorphous

a) The chemical compositions of the synthetic precursors are: 0.5Na_2_O:1SiO_2_:0.7NH_4_Cl:*x*Al_2_O_3_:*y*EmimBr:*z*H_2_O. Reaction occurred at 175°C for 48 h.

Pure phase of the TON-zeolite can be prepared in the composition range of 0.5 Na_2_O:1SiO_2_:0.006–0.025Al_2_O_3_:0.1–0.2EmimBr:0.7NH_4_Cl:4.5–5.0H_2_O through the ILA route. From the results of a series of experiments, which are listed in Table 1, it can be concluded that to successfully prepare the TON-zeolite, the presence of EmimBr is necessary. The absence of ionic liquid leads to a dense α-quartz phase (sample 1, 4, 6 and 9). This indicates the EmimBr plays the role of structure-directing agent. TON-type zeolite has been reported to be prepared by using 1,3-dimethylimidazolium bromide as SDA.[52] The reported SDA (Figure1b) is similar to the ionic liquid used in this contribution (Figure1c). *Wheatley* and co-workers' work has also proved that TON-type isostructural zeolites are crystallized with the aid of a partially hydroxide exchanged ionic liquid.[49] Together with this work, it is reasonable to consider that imidazolium-based compounds are suitable SDAs for the synthesis of the one-dimensional TON-type zeolite.

Meanwhile, the content of aluminum in the synthetic compounds affects the final products as well. Incorporation of aluminum into the framework can result to acidic sites, which are important in the catalysis reaction. The Si/Al ratios in the synthetic starting component should be in the range from 16 to 80. When this ratio is below 16, the product is amorphous (sample 11 and 12). TON type zeolites with Si/Al ratio below 16 are inaccessible through the ILA synthetic route. To the best of our knowledge, all the reported zeolites with TON type structure such as theta-1,[51] ZSM-22,[53] and KZ-2[54] are silica-rich in composition. While the ratio is above 80 to even pure siliceous (sample 2 and 3), impurities, which can be identified as a ZSM-48 structure are observed (Figure2d). The ZSM-48 is a high silica disordered structure (type code of *MRE).[55] The structure of *MRE contains one-dimensional 10-member rings and 5-member ring building units, which are similar to the TON structure. This may be the reason that the competition between the two phases at high synthetic Si/Al ratios.

The Si/Al ratios of the products are analysed by EDX, and the results are listed in Table 2. The Si/Al ratios in the products varied from about 18 to 430, related with the Si/Al ratio in the starting component. The easily control of the Si/Al ratio in such a wide range is helpful for the preparation of catalyst with required acidity.

**Table 2:** The Si/Al ratios for ILA-synthesis of TON-zeolite

No.	Si/Al ratios of synthetic compounds	Si/Al ratios of products
1	16	17.9
2	40	78.4
3	80	426.9

The EmimBr is a hydrophilic compound and would absorb water strongly when employed in industrial procedure. To investigate the effect of absorbed water, small amount of water was added into the synthetic precursor in some occasions. However, no obvious changes were observed by PXRD due to the addition of water (Figure2c). It is also noticed that under moist atmosphere or long grind time, the synthetic compounds transform from powder to paste. This transformation may because of the ionic liquid strongly absorb the water molecules in the air and change the water content in the compound. Both powder and paste are found to be successful precursor for zeolite formation, which implies the ILA synthesis of TON zeolite is a facile, repeatable method and tolerable to the operation environment.

**Figure 3:**
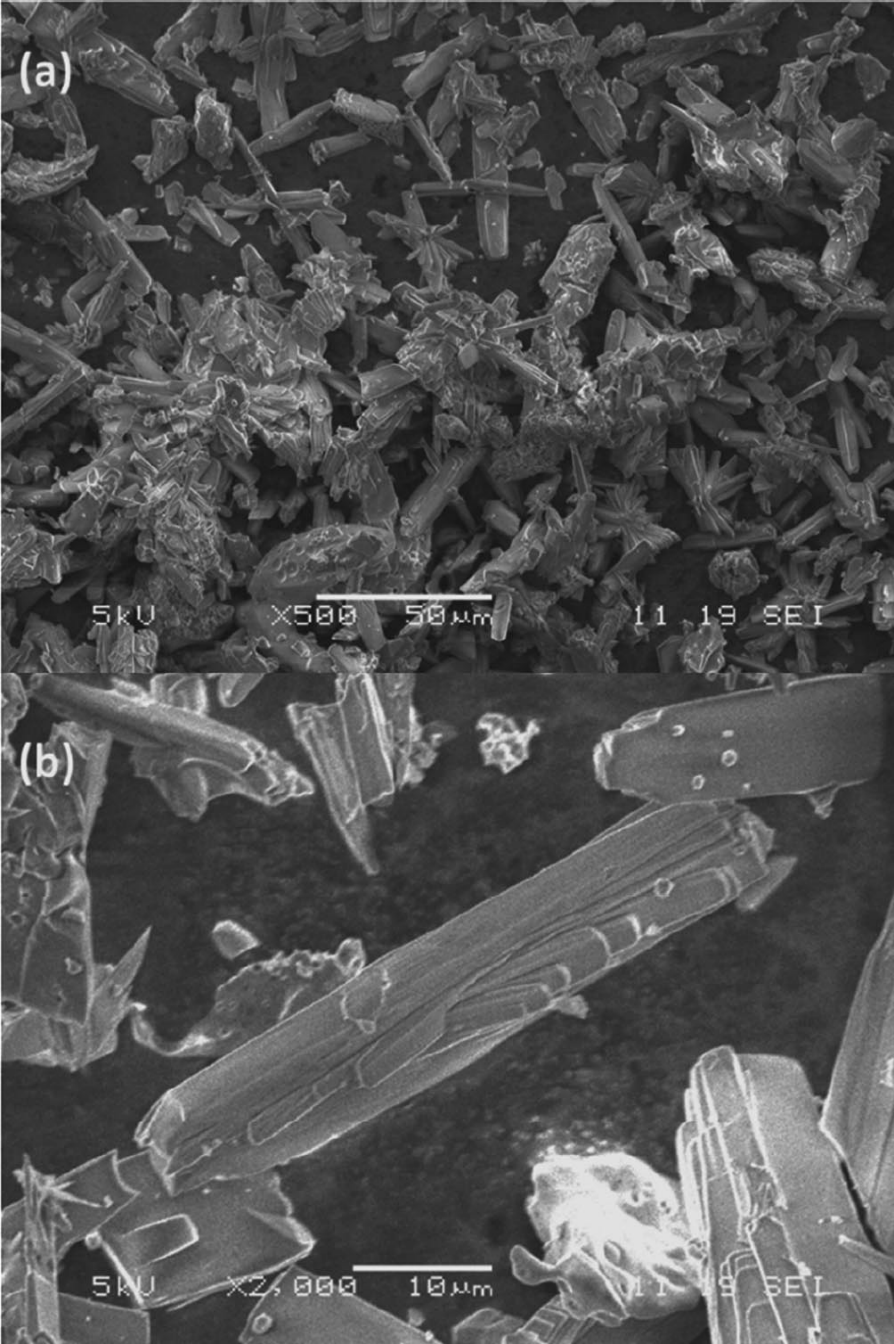
SEM images of as-made TON crystals, which are taken at (a) low magnification (Scale bar is 50 μm) and (b) high magnification (scale bar is 10 μm).

The SEM images of a pure TON zeolite sample (No. 7) are shown in Figure3. The products are prism-shape. The intergrowth morphology of the products (Figure3b) is possibly because of the very high concentration of the tetrahedral species in such an almost “solid-state” system. The particle sizes of the TON-zeolites are in the range of 10–50 μm. It is worth noting that the zeolite crystals, which are synthesized from this solvent free route are usually large.[38] Several zeolite materials were synthesized with the same prism shape but < 5 μm in length.[51,56] Zeolites with appropriate size bigger than 5 μm are needed in liquid continuous flow processes.[57] This ILA synthesis provided a facile route to prepare large zeolite crystals.

**Figure 4:**
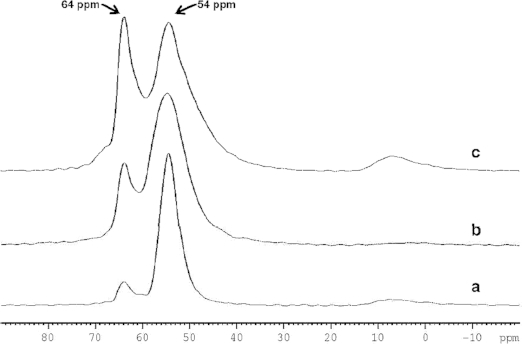
^27^Al MAS NMR spectra of calcined TON-zeolite obtained from (a) sample 5, (b) sample 7, and (c) sample 10.

Solid state NMR can supply information of the local atomic environment of the crystals. The ^27^Al MAS NMR of calcined sample and ^13^C MAS NMR of the as-made sample were investigated. The ^27^Al NMR spectra of the samples with different Si/Al ratios are shown in Figure4. All the spectra exhibit two resonances at chemical shift of 54 and 64 ppm, which are referred to two tetrahedral coordinated central atoms. The relative integrals of each resonance indicate the preferential Al location for the samples with different Al contents: Aluminum atoms firstly incorporated in the framework sites of 54 ppm resonance, then with the increase of Al contents the sites of 64 ppm resonance were gradually occupied.

A minor broad resonance at 6 ppm indicates the existence of a very small amount of octahedral coordinated Al, which may come from the unreacted Al species. The very low intensity of the resonance from the octahedral Al implies that almost all the Al species are in the framework.

**Figure 5:**
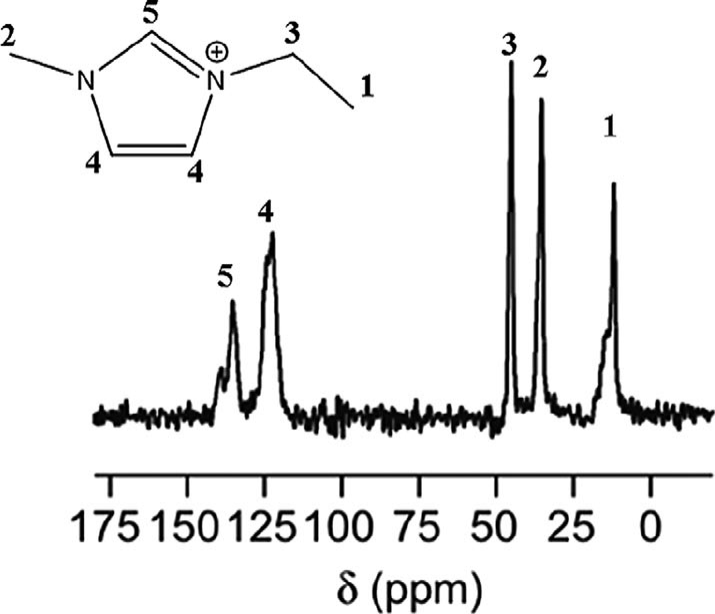
^13^C MAS NMR spectrum of as-synthesized TON-zeolite from sample 7: the inserted figure shows the structure of the Emim^+^ cation and the corresponding carbon atoms to the resonances.

Figure5 shows the ^13^C NMR spectrum of the as-synthesized product from sample 7. The resonances and the corresponding carbon atoms of the EmimBr are observed, indicating the ionic liquid is stable after synthetic procedure and exists in the channel as SDA.

**Figure 6:**
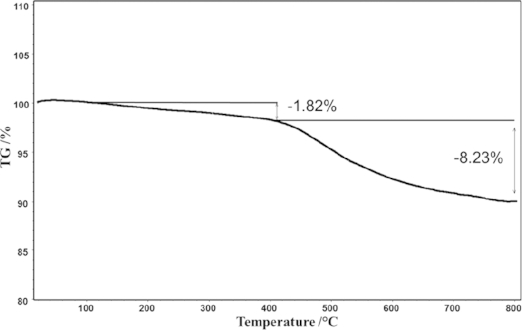
TGA curve of as-made TON-zeolites calcined to 800 °C in air.

Figure6 shows the TGA curve of the as-made sample when heated to 800 °C under air. A weight loss of about 1.8 % in the interval from 100 to 400 °C is attributed to the removal of the adsorbed water molecules in the channels. As the temperature increases to 800 °C, further loss of about 8.2 % is observed. This is considered as the pyrolytic decomposition of the volatile cations, including NH^4+^ and Emim^+^.[58]

**Figure 7:**
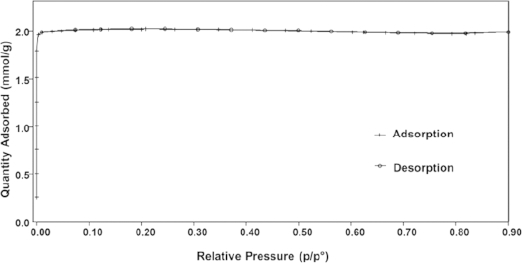
N_2_ isothermal plots of calcined TON-zeolites.

The porous property of the prepared TON zeolites is characterized by nitrogen adsorption (Figure7). The isothermal adsorption curve of the calcined sample is a typical type I adsorption, which indicates the microporous property of the product. The BET surface area of the calcined sample is calculated to be 170 m^2^**·**g^–1^ and the pore volume is 0.07 cm^3^**·**g^–1^. Compared to the reported Theta-1 zeolites, which possesses 0.1 cm^3^**·**g^–1^ pore volume,[51] the slightly decrease may cause by the increasing crystal size.

## Conclusions

In this work an ionic liquid assisted route for the synthesis of aluminosilicate zeolite materials is reported. The zeolite is prepared in an almost solvent-free condition, which reduces the produce of waste water. By the assistance of the most commonly used ionic liquid, EmimBr, an aluminosilicate TON zeolite with the uni-dimensional 10-member-ring channel is synthesized. The ILA synthetic route can prepare zeolites in the sizes of 10–50 μm. The Si/Al ratios, which are important for the catalysis property can be controlled in the range from 18 to 430. By selecting different ionic liquids, other structures of zeolitic materials would potentially be prepared through this route.

## Experimental Section

A typical ionic liquid assisted synthesis procedure is described as follows. 1-Ethyl-3-methylimidazolium bromide (EmimBr) was prepared following the literature.[32] Na_2_SiO_3_**·**9H_2_O (1.4 g, 5 mmol), fumed silica (0.30 g, 5 mmol), and Al(OH)_3_ (0.020 g, 0.3 mmol) were ground in a mortar for 5 min. NH_4_Cl (0.40 g, 7.5 mmol) and EmimBr (0.17 g, 0.9 mmol) were added into the mixture and ground for another 5 min. In the cases of investigating the effect of moist air to the synthesis, small amount of distilled water (0.1 g, 5 mmmol) was added into the mixture. White powder, or in some occasions such as moist environment, white paste could be formed as the synthetic compound. The powder or paste was transferred into a Teflon lined stainless steel autoclave and heated at 175 °C for 2 d. The product was washed with distilled water, filtered, and dried at 90 °C overnight. Calcination was carried out at 550 °C for 6 h to remove the volatile cations such as NH^4+^ and Emin^+^ in the channels. The product weight after calcination was about 0.530 g, and the yield was about 88 % based on silica.

The samples were filled in the glass capillaries of 0.5 mm in diameter for PXRD analysis. The PXRD data were collected with a STOE diffractometer under Cu-*K*_α_ radiation in Debye-Scherrer mode. Thermal gravimetric analysis (TGA) was carried out with a Netzsch TG 209 instrument under flow of air. The sample was heated in alumina crucible at a heating rate of 10 K**·**min^–1^. The morphologies of the product were examined by scanning electron microscopy (SEM) using a Jeol JSM-5600 scanning electron microscope. A tungsten filament is used as the electron source and the operating high voltage was 5 kV. Chemical composition was investigated by Energy Dispersive X-rays (EDX) with an Oxford Inca Energy system at operating voltage of 25 kV. Surface area was examined with nitrogen at 77 K using a Micromeritics ASAP 2020 instrument. The samples were degassed at 110 °C for 8 h. Solid-state NMR spectra were acquired with a Bruker Avance III 600 MHz spectrometer equipped with a widebore 14.1 T magnet. Powdered samples were packed into conventional 4 mm ZrO_2_ rotors. ^13^C spectra were acquired using CP, with a contact pulse (ramped for ^1^H) of 2 ms, and ^1^H decoupling applied during acquisition. For the ^27^Al MAS NMR the MAS rate of 14 kHz was used.
